# Flortaucipir tau PET imaging in semantic variant primary progressive aphasia

**DOI:** 10.1136/jnnp-2017-316409

**Published:** 2017-10-06

**Authors:** Sara J Makaretz, Megan Quimby, Jessica Collins, Nikos Makris, Scott McGinnis, Aaron Schultz, Neil Vasdev, Keith A Johnson, Bradford C Dickerson

**Affiliations:** 1 Frontotemporal Disorders Unit, Department of Neurology, Massachusetts General Hospital and Harvard Medical School, Charlestown, Massachusetts, USA; 2 Center for Morphometric Analysis, Department of Psychiatry, Massachusetts General Hospital and Harvard Medical School, Charlestown, Massachusetts, USA; 3 Center for Alzheimer Research and Treatment, Department of Neurology, Brigham and Women’s Hospital, Boston, Massachusetts, USA; 4 Martinos Center for Biomedical Imaging, Massachusetts General Hospital and Harvard Medical School, Charlestown, Massachusetts, USA; 5 Alzheimer’s Disease Research Center, Department of Neurology, Massachusetts General Hospital and Harvard Medical School, Boston, Massachusetts, USA; 6 Division of Nuclear Medicine and Molecular Imaging, Department of Radiology, Massachusetts General Hospital and Harvard Medical School, Boston, Massachusetts, USA

**Keywords:** brain atrophy, tau PET, primary progressive aphasia, semantic dementia, temporallobe

## Abstract

**Objective:**

The semantic variant of primary progressive aphasia (svPPA) is typically associated with frontotemporal lobar degeneration (FTLD) with longTAR DNA-binding protein (TDP)-43-positive neuropil threads and dystrophic neurites (type C), and is only rarely due to a primary tauopathy or Alzheimer’s disease. We undertook this study to investigate the localisation and magnitude of the presumed tau Positron Emission Tomography (PET) tracer [^18^F]Flortaucipir (FTP; also known as T807 or AV1451) in patients with svPPA, hypothesising that most patients would not show tracer uptake different from controls.

**Methods:**

FTP and [^11^C]Pittsburgh compound B PET imaging as well as MRI were performed in seven patients with svPPA and in 20 controls. FTP signal was analysed by visual inspection and by quantitative comparison to controls, with and without partial volume correction.

**Results:**

All seven patients showed elevated FTP uptake in the anterior temporal lobe with a leftward asymmetry that was not observed in healthy controls. This elevated FTP signal, largely co-localised with atrophy, was evident on both visual inspection and quantitative cortical surface-based analysis. Five patients were amyloid negative, one was amyloid positive and one has an unknown amyloid status.

**Conclusions:**

In this series of patients with clinical profiles, structural MRI and amyloid PET imaging typical for svPPA, FTP signal was unexpectedly elevated with a spatial pattern localised to areas of atrophy. This raises questions about the possible off-target binding of this tracer to non-tau molecules associated with neurodegeneration. Further investigation with autopsy analysis will help illuminate the binding target(s) of FTP in cases of suspected FTLD-TDP neuropathology.

## Introduction

The semantic variant of primary progressive aphasia (svPPA) is a form of PPA with well-characterised, relatively stereotypical clinical features initially manifesting as a fluent, anomic aphasia with prominent semantic memory loss associated with asymmetrical (usually dominant hemisphere) anterior temporal cortical atrophy.^1–4^ In more than 90% of cases with this characteristic clinic-anatomical phenotype, the underlying neuropathology is frontotemporal lobar degeneration (FTLD) with long TDP-43-positive neuropil threads and dystrophic neurites in temporal and frontal cortices (known as TDP-43 type C pathology).^5 6^ Rarely, the primary neuropathology of svPPA is a tauopathy—usually Alzheimer’s disease (AD) or Pick’s disease,^7–9^ or as has recently been reported, globular glial tauopathy.^10^

The advent of tau PET imaging has revolutionised our ability to measure neurofibrillary paired helical filament (PHF) tau pathology in AD, but findings with these ligands in non-Alzheimer tauopathies with straight filament tau are less clear. To date, small in vivo series have been reported using the presumptive tau PET ligand flortaucipir (FTP) in patients with clinical diagnoses of progressive supranuclear palsy, Microtubule-associated protein tau (MAPT)-related FTLD and corticobasal syndrome,^11–13^ suggesting that tracer uptake is present in areas of expected neurodegeneration. However, autoradiographic and biochemical studies of postmortem tissue from patients with non-AD tauopathies have shown weak or no evidence of FTP binding to straight filament tau, and no binding to TDP inclusions.^14–17^

We undertook this study to investigate the uptake, localisation and magnitude of the putative tau PET tracer FTP in a series of patients with svPPA. Since the majority of patients with svPPA have TDP-43 pathology, we hypothesised that the majority of our sample will have non-tau pathology and exhibit FTP signal no different from controls, and have little or no evidence of cortical amyloid plaques. Although far less likely, our sample may include patients with svPPA due to AD or a primary tauopathy. If a patient in our sample has svPPA due to AD pathology, we expected to see evidence of cortical amyloid, and FTP binding localised congruently with atrophy and at a magnitude consistent with what has been observed in other reports of patients with atypical AD.^18 19^ If a patient has svPPA due to a primary tauopathy such as Pick’s disease, we expected little or no evidence of cortical amyloid, and low FTP binding consistent with postmortem findings of low or no FTP affinity for straight filament tau. Alternatively, if FTP binds to a non-specific marker of neurodegeneration, we would expect increased FTP signal in all of the patients with svPPA, co-localised with atrophy.

## Methods

### Participants

#### svPPA Patients

Seven patients with svPPA were recruited from an ongoing longitudinal study being conducted in the Primary Progressive Aphasia Program of the Massachusetts General Hospital (MGH) Frontotemporal Disorders Unit (see table 1). The patients were all right-handed, native English speakers. All patients with svPPA were evaluated using a structured clinical assessment performed by a behavioural neurologist and speech pathologist, as previously described, including rating using the Progressive Aphasia Severity Scale.^1 20^ All participants gave written informed consent in accordance with the institutional review board of the MGH and Partners Healthcare System Human Research Committee.

**Table 1:** Demographic and clinical characteristics of participants

	Age	Sex	Illness duration	PiB	MMSE*	CDR			PASS		BNT
				(FLR DVR)		Global	Behaviour	Language	SOB	Single word	
Case 1	72	F	10 years	1.05	23/30	0.5	0	1	3	1	1
Case 2	66	M	8 years	†	14/30	1	1	2	13.5	2	1
Case 3	71	F	3 years	‡	23/30	0.5	0	1	3.5	1	1
Case 4	54	F	6 years	1.04	24/30	0.5	0.5	0.5	4.5	0.5	2
Case 5	53	M	4 years	1.06	20/30	0.5	1	1	7.5	1	1
Case 6	63	F	6 years	1.07	25/30	0.5	0	1	5	1	3
Case 7	80	M	6 years	1.49	19/30	0.5	0.5	1	7	0.5	4
svPPA n=7	66.0±9.6	4 F	6.6±2.5	1.14±0.19	21.1±3.8						
Control n=20	62.8±8.8	9 F	-	1.09±0.05	29.5±0.6						

Group values for age, PiB, FLR DVR and MMSE are mean ± SD.

*Significant difference between svPPA and control groups, p<0.001.

† Case 2 did not complete PiB PET nor was CSF available.

‡ Case 3 had CSF amyloid beta and phospho-tau levels consistent with the absence of cerebral amyloid plaques and neurofibrillary tangles.

BNT, Boston Naming Test summary score (# correct, spontaneous or with semantic cue); CDR, Clinical Dementia Rating; FLR DVR, distribution volume ratio of FLR regions (ie, frontal, lateral parietal and temporal, and retrosplenial cortices); MMSE, Mini-Mental State Examination; PASS, Progressive Aphasia Severity Scale; PiB PET, Pittsburgh compound B PET; single word, PASS single word comprehension score; SOB, PASS Sum of Boxes score; svPPA, semantic variant of primary progressive aphasia.

Patients were diagnosed with svPPA based on consensus guidelines.^2^ A diagnosis of PPA required progressive deterioration of speech and/or language functions, and that deficits be largely restricted to speech and/or language for at least the first 2 years of the illness.^21^ Each of the patients with svPPA included in this study had prototypical clinical (semantic language impairments) and anatomical (cortical atrophy that was most prominent in the left anterior temporal lobes) characteristics on first assessment, and was at a mild stage of overall clinical impairment at time of scanning. Visual inspection of a clinical MRI ruled out other causes of focal brain damage and in all cases provided imaging support for the diagnosis (anterior temporal atrophy).

#### Controls

Imaging data from 20 age-matched and gender-matched, right-handed, cognitively and neurologically normal controls were included. All 20 individuals underwent detailed clinical and cognitive assessments and were determined to be cognitively normal. Amyloid PET scanning demonstrated minimal or no evidence of elevated amyloid, with low or minimal amyloid defined as [^11^C]Pittsburgh compound B (PiB) distribution volume ratio (DVR) of FLR regions (ie, frontal, lateral parietal and temporal, and retrosplenial cortices)<1.2.^22^ Age, gender, PiB FLR and Mini-Mental State Examination (MMSE) of controls are summarised in table 1.

### Neuroimaging data acquisition and analysis

#### Magnetic resonance imaging

All participants (patients and controls) underwent a 3T MRI scan (Siemens TIM Trio 3.0T, Siemens Medical Systems, Erlingan, Germany) that included acquisition of T1-weighted multi-echo magnetisation prepared rapid acquisition gradient echo (MPRAGE) structural images. The MRI analysis methods employed here have been previously described in detail, including cortical thickness processing and spherical registration to align subjects’ cortical surfaces (FreeSurfer stable release V. 6.0.0, http://surfer.nmr.mgh.harvard.edu).^23^

#### PET

All seven patients also underwent FTP, and five of the seven underwent [^11^C]PiB imaging. Both FTP and [^11^C]PiB imaging were acquired for all 20 control subjects. FTP was prepared at MGH with a radiochemical yield of 14%±3% and specific activity of 216±60 GBq/μmol (5837±1621 mCi/μmol) at the end of synthesis (60 min), and validated for human use.^24^ [^11^C]PiB was prepared as described previously^17^. All PET data were acquired using a Siemens/CTI (Knoxville, Tennessee) ECAT HR+ scanner (3D mode; 63 image planes; 15.2 cm axial field of view; 5.6 mm transaxial resolution and 2.4 mm slice interval). FTP was acquired from 80 to 100 min after a 10.0±1.0 mCi bolus injection in 4×5 min frames. Recent kinetic studies have shown that some regions may not reach steady state by 100 min postinjection^25^; the standard acquisition window has since been increased for prospective subjects to 80–110 min. [^11^C]PiB PET was acquired with an 8.5 to 15 mCi bolus injection followed immediately by a 60 min dynamic acquisition in 69 frames (12×15 s, 57×60 s). PET data were reconstructed and attenuation corrected, and each frame was evaluated to verify adequate count statistics; interframe head motion was corrected prior to further processing.

To evaluate the anatomy of cortical FTP and [^11^C]PiB binding, each individual patient’s PET data set was rigidly co-registered to the subject’s MPRAGE MRI using SPM8 (Wellcome Department of Cognitive Neurology, Function Imaging Laboratory, London). Visual inspection confirmed accurate registration between anatomical and PET volumes. The cortical regions of interest (ROIs) defined by the FreeSurfer (FS) parcellation were transformed into the PET native space and PET data were sampled within each ROI. Similar to previous reports,^19^ FTP specific binding was expressed in FS ROIs as the standardised uptake value ratio (SUVR) using the cerebellar grey matter ROI as a reference. [^11^C]PiB PET data were expressed in FS ROIs as the distribution volume ratio (DVR) with the cerebellar grey ROI as a reference,^19^ where regional time-activity curves (TAC) were used to compute regional DVRs for each ROI using the Logan graphical method applied to data from 40 to 60 min after injection. For surface-based analyses and ROI quantification, PET signal for each subject was sampled along the cortical ribbon and projected to the nearest surface vertex. Primary analyses of PET data were performed without partial volume correction using geometric transform matrix as implemented in FreeSurfer V. 6.0.0; analyses were repeated with partial volume correction as previously described.^26^

#### Neuroimaging data analysis

PET tracer uptake and cortical atrophy were assessed at both the individual patient and group levels. First, each patient’s images were visually inspected. Then we created individual patient cortical surface maps of elevated FTP uptake and cortical thickness compared with the control group to visualise the spatial distribution of increased FTP in comparison to atrophy. For the individual atrophy map, we performed a surface-based, whole-cortex general linear model (GLM) analysis using FreeSurfer,^19^ which compares the thickness of the individual patient’s cortex at each vertex point across the entire cortical mantle with the group of controls. Similarly, we performed an GLM comparing FTP signal in the individual patient at each vertex point with FTP signal in the group of controls. In addition to the individual patient-level maps, we performed group-level GLMs comparing the entire svPPA group with controls, both for cortical atrophy and increased FTP signal relative to controls.

Finally, to further quantitatively compare the magnitude of FTP tracer uptake between patients and controls, we measured FTP signal within 34 FS ROIs per hemisphere from the Desikan-Killiany *et al* parcellation.^27^ We ran independent sample t-tests on five of these ROIs per hemisphere, selected to encompass areas with elevated FTP signal in the svPPA patient group as described below. In addition, we chose the precuneus ROI as a comparison region. To quantify the relationship between FTP signal and cortical thickness within each patient, we extracted mean FTP SUVR with and without partial volume correction, and cortical thickness for each of the 68 ROIs from the Desikan-Killiany parcellation (34 per hemisphere). A standardised t-score was calculated for cortical thickness within each ROI to represent the magnitude of cortical thickness within the ROI, normalised to the healthy control sample ((Patient cortical thickness − µ_controls_) / (σ_controls_× √((*n*+1) / *n*))).^28^ Within each patient, Pearson correlation analyses (IBM SPSS Statistics V. 22) were performed for all ROIs to assess the relationship between FTP signal and cortical thickness t-scores (‘atrophy’), using a method we previously published.^19^

## Results

Five of seven patients were amyloid negative based on amyloid PET, CSF or both. One patient’s amyloid status was unknown due to inability to tolerate acquisition of a PiB scan or a CSF sample. One older patient with svPPA exhibited elevated PiB PET tracer uptake with a spatial distribution and magnitude (PiB FLR=1.49) consistent with the presence of cortical amyloid plaques. There were no significant differences between the control and svPPA groups with respect to age, gender or PiB FLR; the svPPA group had significantly lower MMSE scores. Table 1 provides details.

**Figure 1:**
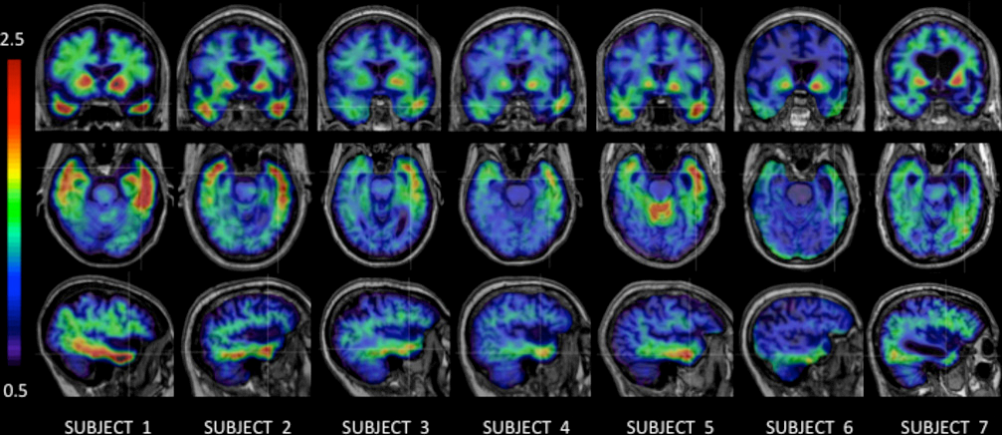
Flortaucipir standardised uptake value ratio overlaid on representative coronal, axial and sagittal MRI slices at levels that illustrate the primary regions of elevated signal in each case, which follows a stereotyped pattern largely co-localised with regional atrophy. These images are not partial volume corrected, and are displayed using a threshold range of 0.5–2.5. Cases 6 and 7 exhibit the lowest signal; case 7 is Pittsburgh compound B PET amyloid positive.

Surprisingly, visual inspection of FTP scans of each individual patient with svPPA showed asymmetrically elevated signal with a spatial distribution consistent with atrophy (see figure 1). The highest cortical signal was in the anterior temporal lobe (left > right) with a variable degree of extension caudally in the ventral temporal cortex. Peak cortical FTP SUVR was ≥2.0 in all but one subject with svPPA. Subject 6 showed a peak FTP SUVR left anterior temporal signal of 1.5.

Slightly lower asymmetrical (left > right) signal was seen in in the inferior temporal gyrus extending caudally in most patients to the caudal hippocampus or beyond, and in the frontoinsula and orbitofrontal cortex. In most patients, the SUVR of this signal was equal to or greater than 1.5. In most patients, elevated signal was also seen in regions within the lateral temporal and parietal cortex, as well as ventrolateral frontal cortex, usually at FTP SUVR equal to or greater than 1.3. The posterior cingulate cortex showed peak FTP SUVR <1.25 in all but three patients (case 7: 1.7; case 1: 1.5; case 2: 1.3).

In the patient with evidence of cortical amyloid plaques (figure 1, subject 7), FTP tracer uptake was localised similarly to the other cases, except that anterior temporal signal was more prominent in the anterior medial temporal region and that a few frontoparietal regions showed slightly elevated signal not seen in the other cases. Consistent with the other six cases, the magnitude of signal in this case was no greater than an SUVR of 2.0 in any region except the rostral ventral striatum in the region of the nucleus accumbens, where it was ~2.2 in the left hemisphere and ~1.9 in the right hemisphere. Most anterior temporal regions with elevated signal showed values in the range of 1.2–1.6. Elevated signal extended caudally in the ventral temporal lobe into posterior fusiform gyrus, greater in the left hemisphere (~1.7–1.8) than right (~1.4). Additionally, elevated signal was seen in left superior frontal gyrus and posterior cingulate cortex (SUVR=1.7). This case also showed the highest signal of all the svPPA cases in the precuneus, slightly above that of controls, as described below.

Both patients and controls showed elevated signal within the basal ganglia, consistent with previous reports.^29 30^ In patients only, elevated signal consistently extended at similar levels into the rostral ventral striatum, anterior to and distinct from the basal ganglia signal present both patients and controls. This rostral ventral striatal signal, in the vicinity of the nucleus accumbens, was highly lateralised (left > right), consistent with lateralisation of cortical atrophy. This striatal signal was the most prominent brain signal in each patient, and peak FTP SUVR was ≥2.0 in voxels within these regions. All subjects showed additional subcortical FTP binding near the substantia nigra of the midbrain, consistent with prior in vivo reports^29^ and postmortem reports of off-target FTP binding to neuromelanin-containing cells.^14 16^

In all patients, elevated FTP signal was present in the cerebral cortical grey matter of the regions described and usually in the subjacent white matter, in some cases with most prominent signal at the grey–white junction. SUV images were inspected, and were confirmed to have the expected uniform, low uptake within the reference ROI. This increases our confidence that elevated FTP SUVR values in the svPPA cases is not due to choice of reference region. The exception to this is one subject with svPPA who exhibited focally increased FTP uptake in the anterior cerebellar grey matter (SUVR=2.1; see figure 1, subject 5). This subject still exhibited high FTP SUVR values (~2.3) in the left anterior temporal lobe, with distribution and magnitude of FTP SUVR values similar to controls in occipital and parietal cortices.

**Figure 2:**
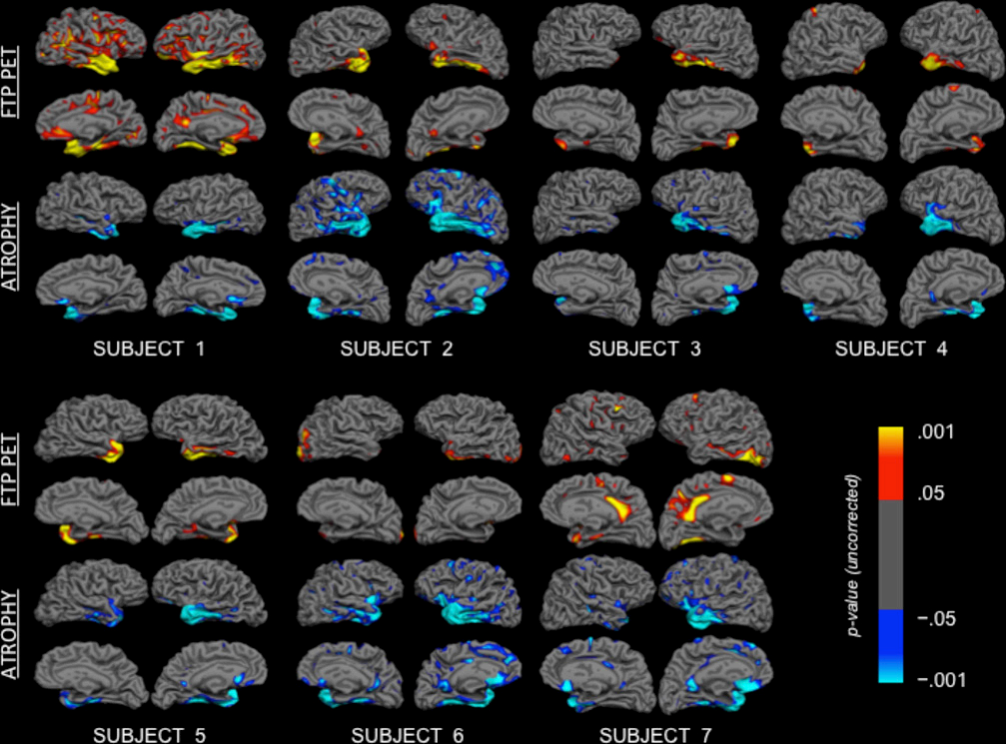
At the individual subject level, the distribution of increased FTP SUVR (red-yellow) and cortical atrophy (blue) are highly overlapping. Surface maps represent vertices with significant differences in measure of interest, as identified with t-tests comparing each subject to the control group. p Values are uncorrected. For each subject, the top two rows show FTP SUVR images, while the bottom two rows show cortical atrophy. FTP, flortaucipir; SUVR, standardised uptake value ratio.

**Figure 3:**
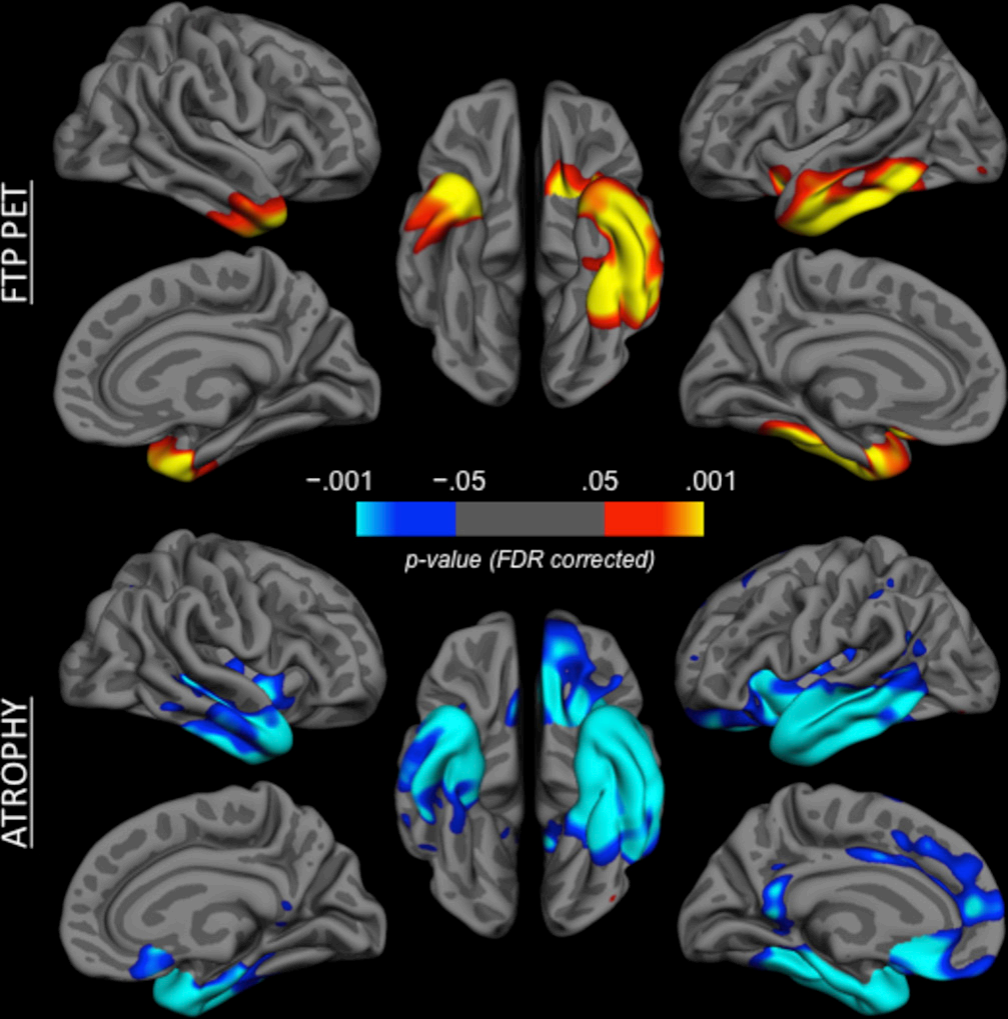
Group comparison of flortaucipir (FTP) standardised uptake value ratio versus controls (top, red-yellow) and cortical atrophy (bottom, blue). Coloured vertices on the cortical surface map indicate areas that are different in the semantic variant of primary progressive aphasia patient group versus the control group. Group comparisons are false discovery rate (FDR) corrected.

Statistical comparison of each individual subject with controls demonstrated that most regions with signal >1.3 described above exhibited signal that was higher than the control group (see figure 2). Furthermore, the localisation of this elevated signal corresponded closely to regional atrophy. Each individual subject’s atrophy map was strikingly similar to the individual subject map of FTP signal. A group map of elevated cortical FTP signal in svPPA compared with controls demonstrated similar findings (see figure 3). The spatial distribution of this elevated signal was highly consistent with the functional connectivity of the temporal pole, as we have previously demonstrated.^1^ Again, at the group level, the atrophy map (see figure 3) strongly overlapped with the map of elevated FTP signal. Few regions demonstrated atrophy without elevated FTP signal.

**Figure 4:**
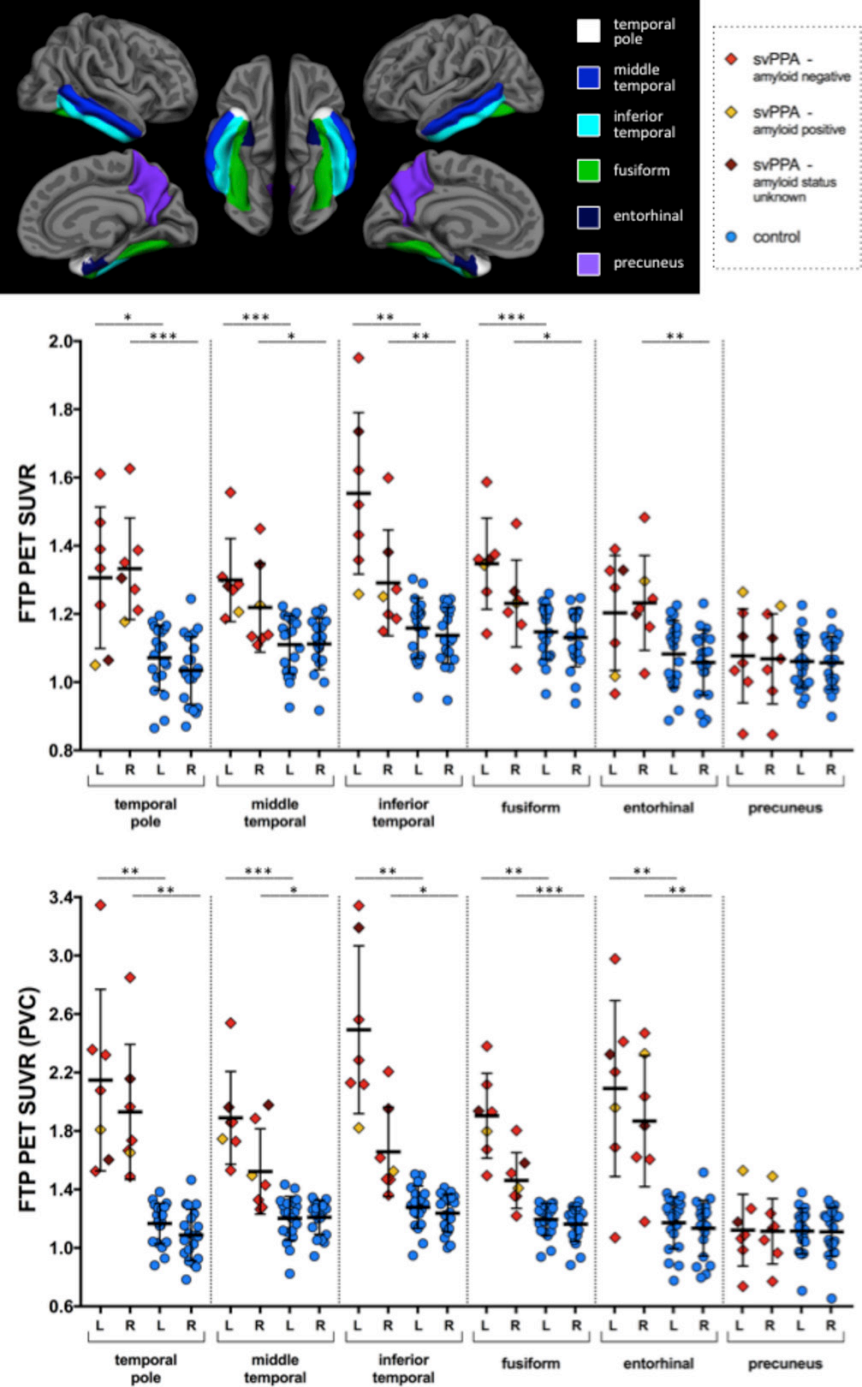
Region of interest measures of FTP SUVR magnitude, with and without partial volume correction. Cortical surface map shows regions of interest from the standard FreeSurfer parcellation. Statistical comparisons are indicated with top left bar comparing left hemisphere regions of interest between svPPA and controls, and lower right bar comparing right hemisphere regions of interest; * p<0.05; ** p<0.01; *** p<0.001. FTP, flortaucipir; SUVR, standardised uptake value ratio; svPPA, semantic variant of primary progressive aphasia.

Within the Desikan-Killiany ROIs we selected for additional analysis, FTP signal was elevated in nearly all patients above the control group in the temporal pole, inferior temporal, middle temporal and fusiform cortex. FTP signal in the entorhinal cortex was elevated in 50% of patients with svPPA. FTP signal in the precuneus was no different in patients with svPPA than in controls. Partial volume correction increases FTP signal substantially in all ROIs except precuneus. See figure 4 for an illustration of the localisation of the ROIs and for FTP signal.

**Figure 5:**
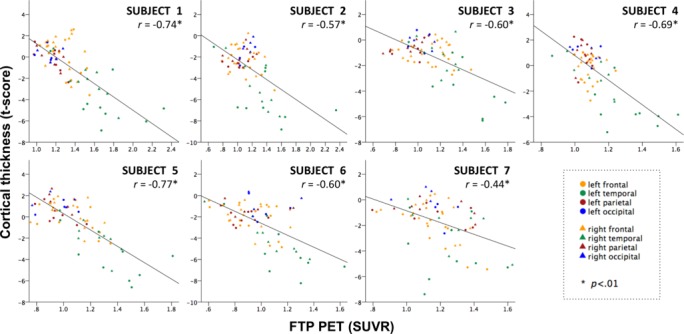
Relationships between cortical atrophy and FTP SUVR measured in 68 cortical regions of interest (ROIs) for each individual patient with svPPA. In each patient, the magnitude of regional FTP SUVR signal is robustly correlated with magnitude of cortical thickness abnormality (cortical thickness t score for each region compared with a group of cognitively normal participants). Each point on each scatterplot represents the values from a single cortical ROI, colour coded by lobe and separated by hemisphere. Note the consistent presence of the temporal lobe regions in the lower right of each scatterplot. FTP, flortaucipir; SUVR, standardised uptake value ratio; svPPA, semantic variant of primary progressive aphasia.

Pearson correlation analyses across the 68 cortical Desikan-Killiany ROIs showed robust relationships between FTP SUVR and magnitude of cortical atrophy within each patient with svPPA (see figure 5). For each patient, correlation between cortical thickness t-score and FTP SUVR showed medium-to-large effects for both uncorrected FTP SUVR (case 1, −0.740; case 2, −0.572; case 3, −0.600; case 4, −0.686; case 5, −0.769; case 6, −0.598; case 7, −0.435) and partial volume corrected (PVC) FTP SUVR values (case 1, −0.779; case 2, −0.627; case 3, −0.643; case 4, −0.733; case 5, −0.771; case 6, −0.568; case 7, −0.661), where ROIs with higher FTP signal also showed more atrophy.

## Discussion

We were surprised to observe that, despite autoradiographic evidence of an absence of binding of FTP in postmortem tissue with FTLD TDP-43 pathology, each individual patient with svPPA in this series demonstrated elevated signal localised asymmetrically in the anterior temporal cortex and other areas of neurodegeneration. The absence of elevated amyloid biomarkers in all but one of these patients indicates that these patients do not likely have AD pathology with PHF tau inclusions. Therefore, the modest elevations in FTP co-localised and correlated with atrophy demonstrate either FTP binding to straight filament tau in the context of a primary tauopathy or non-specific binding co-localised with neurodegeneration in the context of TDP pathology. Based on the field’s collective experience in clinicopathologic studies of patients with this syndrome (>90% with TDP-43 pathology), it seems most likely that our findings point towards FTP binding to a molecule(s) associated with neurodegeneration other than tau. Some investigators have reported that certain forms of TDP-43 pathology bind to thioflavin stains in a manner suggestive of amyloid-like conformational properties,^31 32^ thus offering the possibility that the in vivo signal we observe here is indicative of TDP-43, despite negative FTP autoradiography results with TDP-43 tissue.^14 16^

The spatial distribution of elevated FTP signal in each of the patients with svPPA in the present sample followed a highly stereotyped pattern, consistent with the localisation of typical neuropathology in svPPA or semantic dementia. In each case, the most prominent signal was present in the left temporal pole, inferior temporal gyrus and to a lesser degree middle temporal and fusiform gyri, regions known to be sites of prominent pathology and also known to be closely connected as part of a large-scale network.^1^ To varying degrees in each patient, FTP signal extended caudally into ventromedial and lateral anterior temporal cortical regions. Signal that was similarly localised but lower in magnitude was present in the right temporal pole and ventrolateral temporal cortex.

The localisation of FTP signal was highly similar to the localisation of regional atrophy in each individual patient as well as in group-wide analyses. This is quantitatively demonstrated by the robust correlations within each patient between increased regional FTP signal and more prominent regional atrophy. The localisation and magnitude of FTP are clearly different from the pattern seen in cognitively normal older adults with FTP binding—thought to represent either primary age-related tauopathy or early braak-stage preclinical AD.^29 33^ Furthermore, it is distinct from that of prodromal AD or AD dementia,^18 19 29^ and that of dementia with Lewy bodies.^34^ Yet in most patients with svPPA in this study, the magnitude of signal, even after partial volume correction, is substantially below that seen in patients with likely AD pathology. Interestingly, FTP signal within the precuneus was not elevated in all but one of these patients; it was slightly elevated in the patient with evidence of cerebral amyloid. This may suggest the presence of atypical AD pathology in a patient with svPPA, or dual FTLD-TDP and AD pathology as we have recently reported.^3^ We favour the latter, because the magnitude of FTP signal in this patient is substantially below the magnitude reported in cases of mild dementia driven primarily by AD pathology.

Although spatial resolution limits our ability to reliably distinguish FTP signal in the nucleus accumbens from signal in adjacent striatal structures, all seven patients demonstrated asymmetrically (left>right) elevated signal in the rostral ventromedial striatum in the vicinity of the nucleus accumbens, an area of known pathology in svPPA.^35^ This signal appears to be distinct from high FTP signal seen in more caudal basal ganglia structures that has been reported in both healthy and patient samples.^18 29^

The major limitation of this study is the lack of autopsy follow-up to confirm the neuropathological diagnosis in these cases and the inability to perform tissue studies that would elucidate the possible binding target for FTP in cases with established non-PHF tau pathology. Abundant evidence indicates that the molecule(s) to which FTP binds in these svPPA cases is co-localised with neurodegenerative pathology. Recent data from a different tracer, 18F-THK 5351, indicate that one off-target binding site for that tracer is monoamine oxidase B,^36^ which is expressed in astrocytes and has been proposed as a marker for astrocytosis.^37^ To our knowledge, investigation of this issue with FTP has not been reported. Additionally, the relatively low number of subjects limits the extent to which we can reliably identify group differences. Further studies with FTP and other tau PET tracers need to be performed in patients with svPPA who are ultimately followed to autopsy to elucidate binding targets.
